# The Earliest Known Photo-Documented Rhytidoplasty and Mastopexy (1908): Jules Poullet and the 21st French Surgical Congress

**DOI:** 10.1093/asjof/ojaf156

**Published:** 2025-11-25

**Authors:** Danny J Soares

## Abstract

Aesthetic surgery's present popularity contrasts with its late-nineteenth-century beginnings in commercial “beauty institutes,” where limited, skin-only cosmetic procedures emerged outside the surgical mainstream and left little contemporaneous record. This article reassesses that early period and highlights a neglected primary source: Jules Poullet's 1908 lecture at the 21st French Surgical Congress, published with photographic documentation. Poullet describes a case of pan-facial surgical rejuvenation via cervicofacial rhytidoplasty and upper and lower blepharoplasties, addressing periorbital, midface/temporal, and cervical regions. In the same lecture, he details a mastopexy with periareolar nipple–areola transposition and inframammary reduction, emphasizing scar concealment and appropriate nipple-areola repositioning. Taken together, these contemporaneous reports constitute the earliest known published, photographically documented accounts of both facelifting and mastopexy, shifting the commonly cited timelines by more than a decade. Poullet's contributions, though a small part of his broader surgical career, anticipate core principles of modern facial and breast rejuvenation and clarify the early history of cosmetic surgery before it gained professional recognition.

The ubiquity of aesthetic surgery today stands in sharp contrast to its late-nineteenth-century origins, when cosmetic surgical procedures were stigmatized and often subjected to professional censure.^1-3^ Born out of a commercial sphere of “beauty institutes” and other pseudo-medical ventures that purveyed unproven treatments, early aesthetic medicine struggled for professional legitimacy.^4,5^ It was within this milieu that the earliest, true surgical rejuvenation efforts first appeared, often via limited rhytidoplasties performed by non-specialized physicians interested in harnessing the rejuvenating power of surgery.^6,7^ Nonetheless, because most early cosmetic surgeons left little to no written record, the origins of this core procedure still remain obscure. Accordingly, in this manuscript, we focus on the few surgeons who went on record before the specialty gained broad acceptance, and whose voices helped to steer cosmetic plastic surgery away from a world of unlicensed “beauty culturists” of the late 19th century to the medically accountable specialty of today. It is within this context that the contributions of Dr. Jules Poullet, once lost in time, are herein described in their full significance (Figure 1).^8^

**Figure 1.**
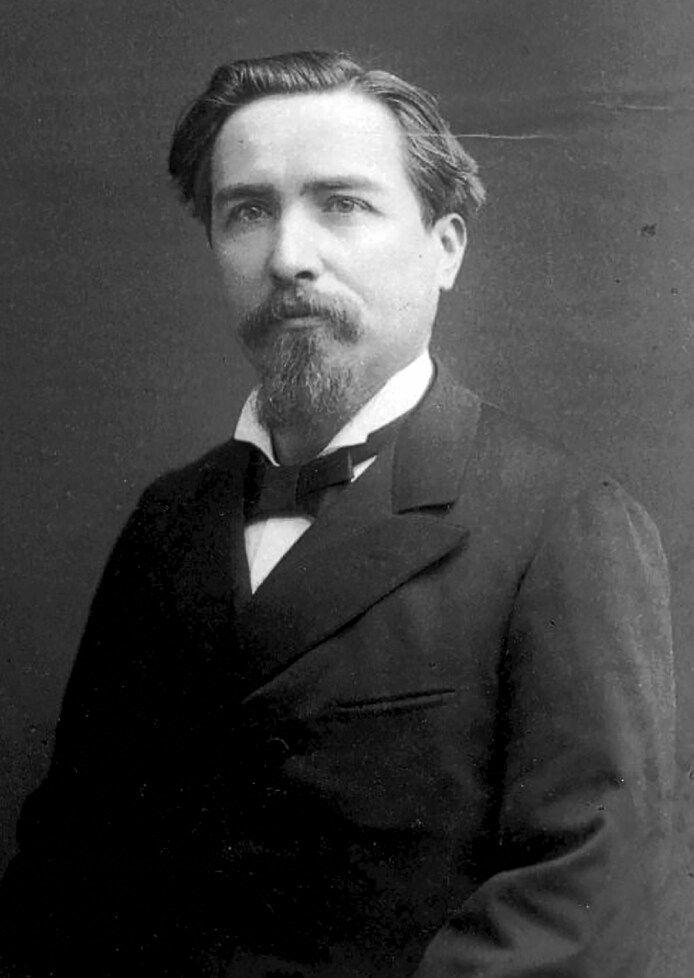
Doctor Jules Poullet (ca. 1881). Dr. Poullet was an early figure in surgical facial and breast rejuvenation; his 1908 presentation at the 21st French Surgical Congress helped bring cosmetic surgery, in particular, rhytidoplasty and mastopexy, into the professional discourse. *Courtesy of Lucien Chabuel, great-grandson of the subject (private collection)*.

## FROM BEAUTY INSTITUTES TO SURGICAL PRACTICE: EARLY RHYTIDOPLASTY

**Figure 2.**
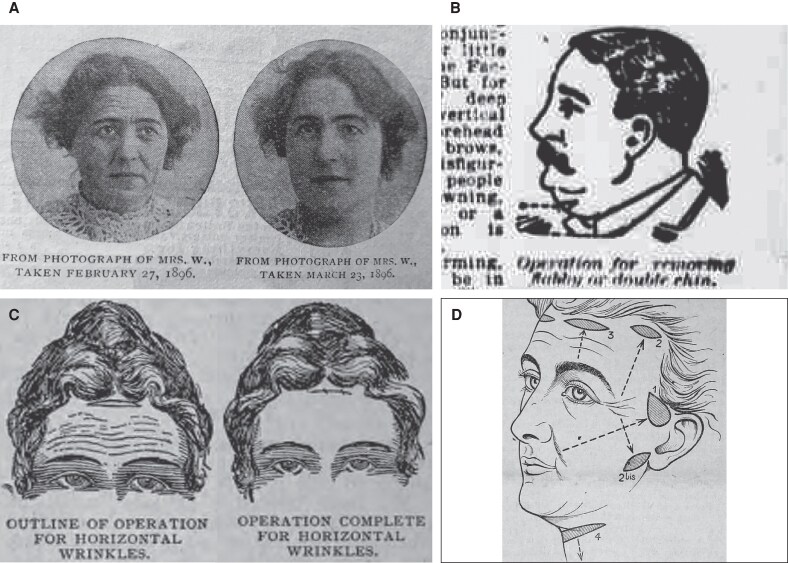
Earliest known print advertisement photographs and illustrations of rhytidoplasty outcomes and technique, from the late 19th century. The late 19th century saw a shift toward the adoption of surgical interventions for cosmetic purposes. (A) An 1896 advertisement by the John H. Woodbury Dermatological Institute showing before and after photographs of a facial rhytidoplasty. From *How faces are transformed* [advertisement]. McClure's Magazine; November 1896; Vol 8, Issue 1, advertising supplement, p. 2. Public domain; Google-digitized; reproduced from HathiTrust Digital Library. (B) Frontal rhytidoplasty diagram from Woodbury Institute promotional booklet, the earliest known reference to a rhytidoplasty. From Woodbury JH. *What Dermatology Has to Do With Beauty: How to Remove All Imperfections of the Skin*. New York, NY: Fless & Ridge Printing Co; 1894. Public Domain. U.S. National Library of Medicine Digital Collections. (C) Advertisement inset from an 1895 magazine showing a submental approach to anterior neck contouring. From *A New Wrinkle: Science Tells How to Remove the Old Ones*. The Evening World (New York, NY), January 16, 1895, p. 6. Public Domain. U.S. National Library of Medicine Digital Collections. (D) First known medical publication outlining rhytidoplasty technique diagrams by Passot in 1919. Despite its significance in the medical literature, most of the incisions outlined in the article had been previously described in print advertisement materials by the Woodbury Institute 20 years earlier. From Passot R, *La chirurgie esthétique des rides du visage*, La Presse Médicale 1919;27(27):258-261. Public Domain. Digitized by the Bibliothèque interuniversitaire de Santé.

The end of the 19th century ushered in an era of intense transformation, marked by health reform and women's suffrage movements, alongside breakthroughs in electrical engineering, transportation, and communication.^9^ This modernization broadened public attention to health and beauty and, crucially, furnished the means for a new repertoire of cosmetic therapies.^10^ The ensuing demand for rejuvenating treatments saw a concomitant increase in the number of “beauty doctors”—unlicensed, non-medical practitioners—leveraging a quasi-clinical setting to peddle a myriad of “wrinkle cures,” from tonics and lotions to facial massaging.^1^ With increasing competition, practitioners devised ever more invasive methods, integrating novel elements, such as electrical stimulation/ablation, resurfacing agents, and even toxic compounds.^11-13^ Unbound by any regulatory constraints and propelled by grandiose claims, these commercial practices initially saw widespread interest; yet mounting litigation over lack of efficacy and treatment-related complications drew sustained negative press, and many operators were increasingly branded as “quacks”.^3^ In response, some beauty institutes sought to bolster credibility, efficacy, and safety by partnering with licensed medical practitioners—particularly dermatologists and surgeons.^14^ As surgical techniques entered the repertoire, clinics could, for the first time, offer more dramatic yet plausibly natural results, even as limited durability and scarring marred the procedure's immediate success (Figure 2A, B).^15,16^ In this early environment, the procedure that would evolve into facelifting took shape as a limited excisional rhytidoplasty, featuring a skin-only resection with minimal undermining, intended to reduce localized laxity and soften conspicuous folds (Figure 2C). Incisions were strategically placed to conceal scars and target individual regions, such as the forehead, lateral canthal region, cheek and jowl, and submentum.^14,16,17^

**Figure 3.**
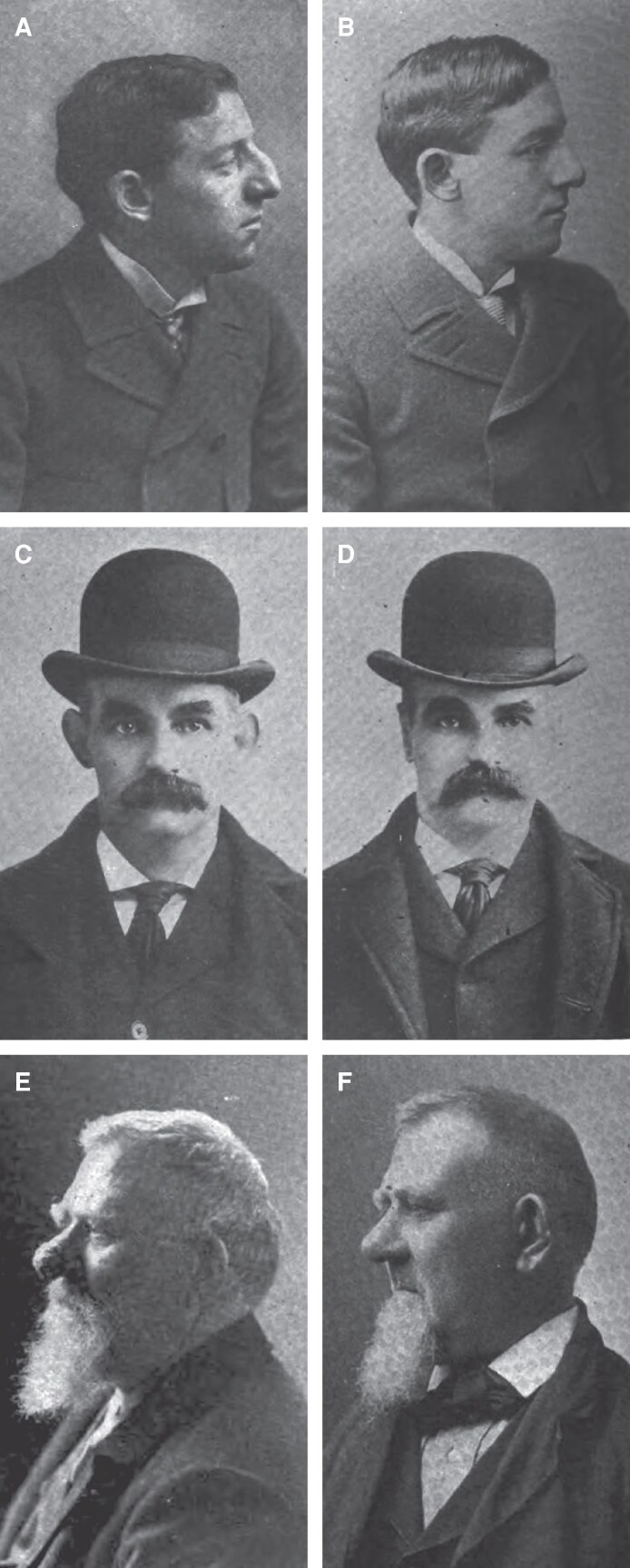
Cosmetic surgical outcome photographs from print advertisement materials, late 19th century. The use of before and after photographs to advertise cosmetic surgery was popularized by the Woodbury Institute, which employed licensed surgeons to perform these cosmetic treatments. (A) Photograph of a patient before and (B) after cosmetic rhinoplasty. (C) Before and after (D) photographs of an otoplasty patient. (E) Male patient before and (F) following ablation of rhinophyma. From *The wonders of modern surgery. Shapely features, Unblemished complexions* [advertisement]. Broadway Magazine; 1899. Public domain. Google-digitized; reproduced from HathiTrust Digital Library.

The John H. Woodbury Dermatological Institute, founded in 1889 in New York City, became emblematic of this turn toward surgical adoption by beauty practices. Led by John Woodbury, a chiropodist and soap impresario, the institute rapidly integrated physician staff to accommodate these novel surgical procedures, growing and expanding into multiple U.S. cities. While Woodbury's technical involvement in the development of these procedures is unclear, evidence suggests that his role within the business was largely executive and promotional following its incorporation in 1890. Although he acted as the institute's figurehead, even going so far as branding himself a “dermatologist,” Woodbury had come under investigation by the New York Medical Society in 1890 for practicing without a license and was ultimately prosecuted by the State of New York in 1891.^18,19^ Court records show, based on testimony by the Institute's own president since 1892, that Woodbury did not treat patients, and that all of the clinical work was carried out by employed surgeons.^20^ It is at this stage of the Institute's existence, from 1892 to 1911, that diagrams of surgical procedures and photographic depictions of surgical outcomes begin to appear in advertising materials (Figure 3).^15,21^

The Woodbury Institute's surgical procedures therefore likely rested with staff doctors, as many as 25 of them, with most of them going unnamed or only occasionally listed in directories and advertisements rather than the scientific press.^14,15,22^ Among those identified are Leonard F. Pitkin, MD (also a founding shareholder) and Thomas M. Acken, MD (New York); Frank T. Brough, MD (Boston); Louis T. Gruel, MD (Philadelphia); Andrew L. Nelden, MD (shareholder, Chicago), John H. Wehrly, MD (St. Louis), and even, for a brief period, influential New York surgeon Frederick S. Kolle, MD.^22,23^ Although John Woodbury helped catalyze the medicalization of aesthetic care, it was almost certainly these clinicians whose work populated the Institute's photographs and brochures from 1892 until its closure in 1911. The efforts of these surgeons marked the passage from quackery to professional medical practice, lending some legitimacy to cosmetic surgery. Nonetheless, despite the rapid integration of surgery into beauty practices, the subspecialty would remain professionally unrecognized in the field of surgery for several decades.

## THE RHYTIDOPLASTY GAINS PROFESSIONAL RECOGNITION

**Figure 4.**
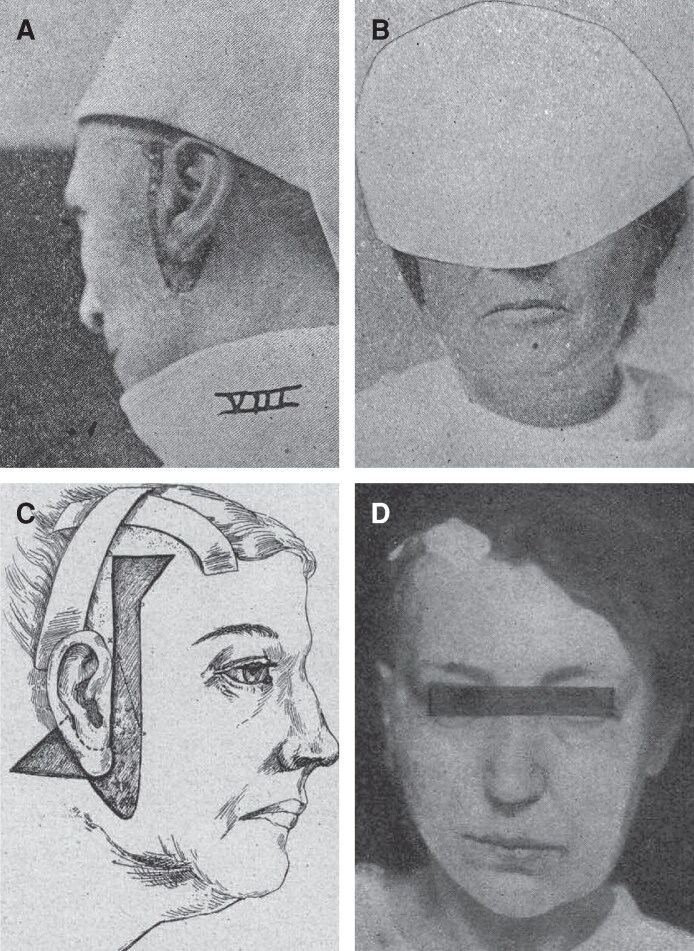
Previous earliest-known surgical photographs of facelift patients published in the medical literature. Prior to the present manuscript, the earliest known photographic depictions of facelifting technique and outcomes were attributed to Adalbert Bettman, MD (1920) and Jacques Joseph, MD (1921). (A, B) Surgical photographs of facelift incision and facelift patient from Bettman's 1920 article, with right-side-only surgical outcome, left face still untreated. From Bettman AG, *Plastic and cosmetic surgery of the face*. Northwest Medicine; 1920;19(8):205-209. Public domain. Digitized by the Historical Medical Library of The College of Physicians of Philadelphia; reproduced via Internet Archive. (C) Technical diagram of facelift incision, and (D) surgical photograph of facelift patient, from Joseph's 1921 article, showing right-side-only result, left face still untreated. From Joseph J. *Hängewangenplastik (Melomioplastik)*. Dtsch Med Wochenschr. 1921;47(11):287-288. doi:10.1055/s-0028-1140504. Accessed August 25, 2025. Public domain. Google-digitized; reproduced from HathiTrust Digital Library.

Despite the rapid ascent of facial rejuvenation in commercial print advertising between 1895 and 1900, the rhytidoplasty remained largely absent from the medical literature for two decades, leaving behind a cosmetic “dark age” in the surgical record. Despite seminal publications on plastic surgery by Miller in 1907/08, Holländer in 1909/12, Lexer in 1910, and Kolle in 1911, the rhytidoplasty still evaded objective description, with Miller disavowing such operations, Kolle favoring volume correction, and Holländer/Lexer offering little operative detail or failing to describe the operation altogether, respectively.^24-28^ It was not until 1919, according to historical narrative, that the rhytidoplasty entered the medical literature through the published works of French surgeons Dr. Raymond Passot and Dr. Julien Bourguet, preempting the cosmetic surgical awakening of the “roaring 20's” that would soon follow.^29,30^ In his publication, Passot described basic elliptical excisions that were similar to those advanced by the Woodbury Institute but served as a published starting point against which subsequent innovations would be measured.^14,29^ Accordingly, Passot's article was quickly followed by other publications, notably by American surgeon Adalbert Bettman (1920) and German surgeon Dr. Jacques Joseph (1921), who both described an extended, peri-auricular incisional approach akin to the modern facelift, and included dramatic split-face before and after photographs to illustrate the procedure's potency (Figure 4).^31,32^ Subsequent contributions by Dr. Suzanne B. Noël (1926), Dr. Otto H. Barnes (1927), and others, consolidated the operation's professional standing and catalyzed further advancements that eventually led to the procedures in use today.^33-35^

### Jules Poullet and the Earliest Known Medical Publication on Rhytidoplasty

Post 1920s, the legitimatization of the facelift procedure incited a flurry of “first-to-facelift” claims by prominent surgeons who, often retroactively, recounted their prescience, such as Holländer's 1932 account of a 1901 facelift, or Lexer's similar 1931 claim.^36,37^ However, even allowing for these recollections, the evidence shows that limited excisional rhytidoplasties were already being performed in the 1890s. By the usual standards of surgical priority—ie, timely presentation, publication to peers, adequate technical detail, and visual documentation—credit belongs to those who put their work on record, especially contemporaneously. On this basis, Jules Poullet's lecture on cosmetic surgery of the face and breast, presented at the 21st French Surgical Congress in 1908, stands as the earliest detailed, published account of a facelift, complete with patient photographs, pushing the historical timeline back by more than a decade.

Pierre-Jules (Jules) Poullet (1842-1917) was born in Lyon, France. He earned his medical doctorate through the Faculty of Medicine of Montpellier in 1866, receiving his surgical training as an intern for the Lyon Hospital system, where he served as prosector between 1864 and 1866. He became head of obstetrics in 1881 and was appointed clinical director of obstetrics at the Lyon Faculty of Medicine by ministerial decree in 1886, where he also served as instructor. During the Franco-Prussian War, he led the *Little Sisters of the Poor* ambulance near Lyon (1870-71) and was later named Officier d’Académie (1891), a national honor for service to education. He published extensively in obstetrics and gynecology and general surgery, pioneering quantitative tocography for the measurement of uterine forces during labor and developing novel hernia repair and nephropexy techniques (Table 1). A prolific instrument designer, he advanced the theory and practice of obstetrical forceps and introduced improved models.^38^ In 1891, he founded the Saint-Louis Surgical Institute in Lyon, where he delivered surgical care ranging from obstetrics to cosmetic surgery.

**Table 1.** Jules Poullet's Medical Publication Topics, by Date

1864	[On the Rupture of the Pelvic Joints During Childbirth]
1866	[Research on heart clots: Thesis presented and publicly defended at the Faculty of Medicine of Montpellier]
1875	[On the Sericeps (silk forceps) (read at the Société de chirurgie, 7 Apr 1875)]
1879	[Obstetrics and Gynecology Abroad (memoir to the Société nationale de médecine de Lyon)]
1879	[Velamentous implantation of the cord considered as a cause of premature rupture of the membranes]
1880	[Fetal hydrocephalus in its relationship to pregnancy and childbirth]
1880	[The Tocograph: Applying the Graphic Method to Labor]
1881	[Flexible Forceps with Independent Tractions (Société nationale de médecine de Lyon)]
1881	[Artificial Induction of Labor in Cases of Prolonged Pregnancy (society communication)]
1882	[Report to the National Society of Medicine on the Candidacy of Professor Wasseige (Liège)]
1883	[The Various Types of Forceps (thesis, Paris; 228 pp., 80 figs)]
1884	[Principles on Which the Construction of a Forceps Should Rest (Archives de tocologie)]
1887	[Oblique Applications of Obstetric Forceps: Angular Forceps]
1894	[Radical cure of hernias, even in the elderly, by the fibro-periosteal flap method]
1895	[On a new nephropexy operation: communication made at the Congress of Swiss Physicians in Lausanne]
1897	[Healing through new and scientific methods in medicine and surgery accessible to all]
1908	[Tendinous Hysteropexy (French Surgical Congress, 6 Oct)]
1908	[Cosmetic Surgery: Face, Breast, Subcuticular Suture. (French Surgical Congress, 10 Oct)]
1916	[Pharmacodynamics and Clinical Applications of Fandorine Therapy]

**Figure 5.**
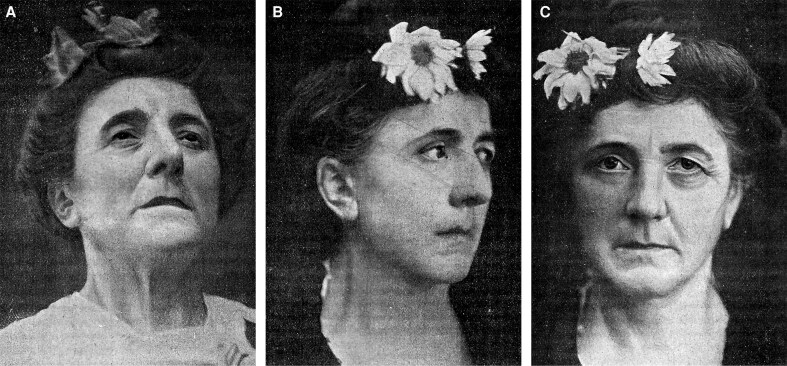
Current earliest-known photographs of a facelift patient published in the medical literature, by Dr. Jules Poullet. Published in 1908 in the proceedings of the 21st French Surgical Congress, Dr. Poullet's lecture described the ageing process and the surgical treatment of facial ageing for each facial region. (A) A 52-year-old female prior to undergoing any treatment. (B) The same patient, following a midface/temporal rhytidoplasty, submental lift, and upper and lower blepharoplasties, post-operative time unknown. (C) Frontal photograph of the same patient with right-side-only surgical outcome, with the left side untreated, showing multi-level facial rejuvenation effect on the right side. From Poullet J. *Présentation de malades: chirurgie esthétique (visage, sein); suture sous-cutanée*. In: Procès-verbaux, mémoires et discussions. 21e Congrès français de chirurgie; Paris, France; 5–October 10, 1908. Paris: Félix Alcan; 1908:992-999. Public domain. Google-digitized; reproduced from Google Books Library Project.

In 1908, toward the end of his career, Dr. Poullet delivered two lectures at the 21st French Surgical Congress, in Paris, titled “Tendinous Hysteropexy” and “Cosmetic Surgery: face, breast, and subcuticular sutures.” In the latter, he chronicles his 6-year experience with the surgical correction of age-related soft-tissue ptosis of the face and breast. Point by point, he describes the multifactorial nature of ageing, emphasizing diminished tissue elasticity, repetitive muscular contraction, volume loss, gravity, and hormonal influences. He argues that folds arise where lax tissue buckles against fixed ligamentous insertions, exemplified by the nasolabial fold. Although succinct in technical detail, Poullet carefully presents a right-side–only rejuvenation outcome in a 52-year-old patient who underwent midface/temporal rhytidoplasty, submental lift, and upper and lower blepharoplasties, with incredible outcomes for the time (Figure 5). For the cervical component, he describes extending the rhytidoplasty incision into the post-auricular region and along the occipital hairline, the earliest documented description of this posterior extension in plastic surgery, previously attributed to Bettman and Joseph.^35^ His multi-procedural approach to facial rejuvenation mirrors modern approaches, delivering immediate, striking outcomes, even by today's standards.

### Also From Poullet's 1908 Lecture: Earliest Known Mastopexy With Nipple–Areola Transposition

**Figure 6.**
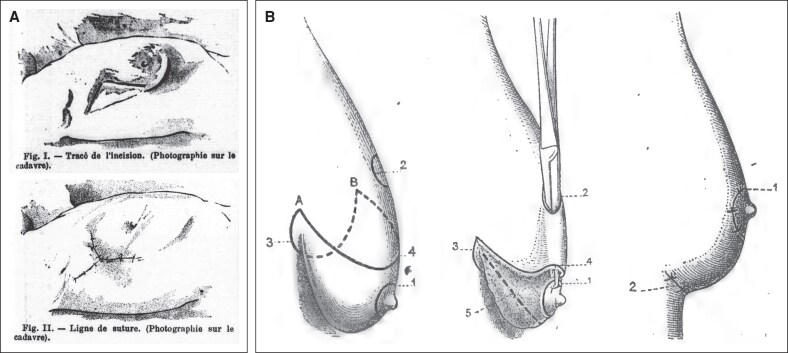
Diagrams of early published mastopexy techniques, by Verchère (1898) and Passot (1925). Early cosmetic mastopexy techniques originated in the late 19th century, featuring simple techniques based on cutaneous excisions. Later on, technical modifications employing nipple-areola-complex (NAC) transposition with inferior reduction were advanced by surgeons like Erich Lexer, Raymond Passot, and Jacques Joseph, among others. (A) Technical illustrations from Verchère's 1898 mastopexy article, employing a lateral/axillary wedge excision. This technique resulted in superolateral distortion of the breast and NAC. From Verchère F. *Mastopexie latérale contre la mastoptose hypertrophique*. La Médecine moderne. 1898;9:540-541. Public Domain, digitized by Bibliothèque nationale de France/Gallica. (B) Mastopexy technique sequence by Passot, from 1925, previously considered to be one of the earliest known diagrams depicting a mastopexy employing NAC transposition and inframammary fold incision. From Passot R, *La correction esthétique du prolapsus mammaire par le procédé de la transposition du mamelon*. Presse Med. 1925;33:317-318. Public domain. digitized by BIU Santé (Medica); reproduced via Internet Archive.

**Figure 7.**
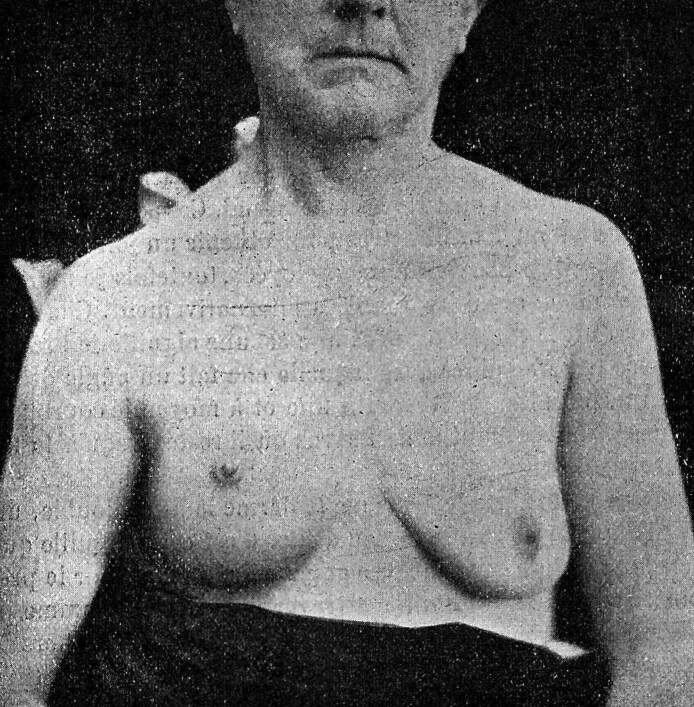
Current earliest-known published photograph of a mastopexy with nipple-areola complex transposition patient, by Dr. Jules Poullet, circa 1908. The photograph depicts a 52-year-old patient (the same patient depicted in the facelift photographs) following a right-side-only mastopexy with nipple-areola complex (NAC) transposition. This technical description represents the current earliest-known photograph of a mastopexy with peri-areolar incision, NAC transposition, and inferior reduction and inframammary closure, preceding the previous known publications by Lexer and Passot by 15 years. From Poullet J. *Présentation de malades: chirurgie esthétique (visage, sein); suture sous-cutanée*. In: Procès-verbaux, mémoires et discussions. 21e Congrès français de chirurgie; Paris, France; 5–October 10, 1908. Paris: Félix Alcan; 1908:992-999. Accessed August 25, 2025. Public domain. Google-digitized; reproduced from Google Books Library Project.

By 1908, the surgical techniques for management of ptotic and/or hypertrophic breasts were few and crude, largely limited to wedge resections with a high cicatricial burden and frequent contour distortion.^39^ Surgical approaches at the time included Pousson's 1897 superior crescent excision, Verchère's 1898 lateral/axillary wedge suspension, and Morestin's 1907 inframammary excision.^40-42^ Despite occasional attempts at parenchymal suspension, these methods were commonly marred by unacceptable scarring, loss of shape and contour, or malposition of the nipple–areola complex (NAC).^39^ According to the prevailing narrative, true mastopexy with NAC transposition did not appear until the 1920s, first by Lexer in 1923 (described by Kraske) and then by Passot, Dartigues, and Joseph in 1925 (Figure 6).^43-46^ These early techniques employed the traditional periareolar incision for NAC transposition, combined with inferior dermoglandular excision and an inframammary closure. Remarkably, Jules Poullet describes a similar mastopexy technique in his 1908 lecture, employing NAC transposition and an inferiorly-based reduction 15 years prior to currently recognized surgical history, with excellent results for the time period (Figure 7).^8^ Though Poullet's wide skin undermining was soon disfavored due to increased ischemic risk, his emphasis on concealed scars, reliable NAC repositioning, and inframammary closure anticipated key tenets of modern mastopexy.^47^

Taken together, these findings position Pierre-Jules Poullet as a key contributor in the early cosmetic-surgery historical record. Although he died in 1917, just before the field's professional and social acceptance accelerated, his contemporaneous documentation of indications, technique, and results arrived at a pivotal moment in the development of cosmetic plastic surgery, more than a decade prior to accepted timelines for rhytidoplasty and mastopexy. Even though cosmetic surgery comprised only a portion of his prolific career, these contributions demonstrate his range as a surgeon-educator and support his inclusion in the specialty's early canon. As proceedings, theses, and society transactions are increasingly digitized, additional contributions and contributors will likely come to light, further clarifying the field's early formative period. For now, Poullet's publications serve as a reliable reference point for understanding the specialty's origins.

## CONCLUSIONS

Our evolving understanding of the early history of plastic surgery requires the piecing together of obscure manuscripts, once lost in time, by individuals whose dedication to the specialty often went professionally unrecognized. Although early methods were rudimentary by today's standards, they established the framework for a technical approach that enabled modern advances in surgical technique. As these sources are integrated, several milestones typically shift earlier than commonly cited. Notably, Pierre-Jules Poullet's 1908 congress report, documenting a rhytidoplasty with panfacial rejuvenation, and a mastopexy with periareolar nipple–areola complex (NAC) transposition, moves the first published accounts of both procedures back by more than a decade. His attention to scar concealment, cervical contour restoration, and reliable NAC repositioning anticipated core principles of current practice. Recognizing Poullet's role refines the historical record and assigns deserved credit to an early visionary whose work helped the specialty attain professional recognition.
